# Pineapple SWEET10 is a glucose transporter

**DOI:** 10.1093/hr/uhad175

**Published:** 2023-04-12

**Authors:** Beenish Fakher, M Arif Ashraf, Lulu Wang, Xiaomei Wang, Ping Zheng, Mohammad Aslam, Yuan Qin

**Affiliations:** State Key Laboratory for Conservation and Utilization of Subtropical Agro-Bioresources, Guangxi Key Lab of Sugarcane Biology, College of Agriculture, Guangxi University, Nanning 530004, Guangxi, China; College of Life Sciences, Key Laboratory of Genetics, Breeding and Multiple Utilization of Crops, Ministry of Education, Fujian Provincial Key Laboratory of Haixia Applied Plant Systems Biology, College of Agriculture, Fujian Agriculture and Forestry University, Fuzhou, Fujian 350002, China; Department of Biology, Howard University, Washington DC 20059, USA; State Key Laboratory for Conservation and Utilization of Subtropical Agro-Bioresources, Guangxi Key Lab of Sugarcane Biology, College of Agriculture, Guangxi University, Nanning 530004, Guangxi, China; College of Life Sciences, Key Laboratory of Genetics, Breeding and Multiple Utilization of Crops, Ministry of Education, Fujian Provincial Key Laboratory of Haixia Applied Plant Systems Biology, College of Agriculture, Fujian Agriculture and Forestry University, Fuzhou, Fujian 350002, China; Horticulture Research Institute, Guangxi Academy of Agricultural Sciences, Nanning Investigation Station of South Subtropical Fruit Trees, Ministry of Agriculture, Nanning 530004, China; College of Life Sciences, Key Laboratory of Genetics, Breeding and Multiple Utilization of Crops, Ministry of Education, Fujian Provincial Key Laboratory of Haixia Applied Plant Systems Biology, College of Agriculture, Fujian Agriculture and Forestry University, Fuzhou, Fujian 350002, China; College of Life Sciences, Key Laboratory of Genetics, Breeding and Multiple Utilization of Crops, Ministry of Education, Fujian Provincial Key Laboratory of Haixia Applied Plant Systems Biology, College of Agriculture, Fujian Agriculture and Forestry University, Fuzhou, Fujian 350002, China; Donald Danforth Plant Science Center, Saint Louis, MO 63132, USA; College of Life Sciences, Key Laboratory of Genetics, Breeding and Multiple Utilization of Crops, Ministry of Education, Fujian Provincial Key Laboratory of Haixia Applied Plant Systems Biology, College of Agriculture, Fujian Agriculture and Forestry University, Fuzhou, Fujian 350002, China

## Abstract

SWEET transporters are a unique class of sugar transporters that play vital roles in various developmental and physiological processes in plants. While the functions of SWEETs have been well established in model plants such as *Arabidopsis*, their functions in economically important fruit crops like pineapple have not been well studied. Here we aimed to investigate the substrate specificity of pineapple SWEETs by comparing the protein sequences of known glucose and sucrose transporters in *Arabidopsis* with those in pineapple. Our genome-wide approach and 3D structure comparison showed that the *Arabidopsis* SWEET8 homolog in pineapple, AcSWEET10, shares similar sequences and protein properties responsible for glucose transport. To determine the functional conservation of AcSWEET10, we tested its ability to complement glucose transport mutants in yeast and analyzed its expression in stamens and impact on the microspore phenotype and seed set in transgenic *Arabidopsis*. The results showed that AcSWEET10 is functionally equivalent to AtSWEET8 and plays a critical role in regulating microspore formation through the regulation of the *Callose synthase5* (*CalS5*), which highlights the importance of SWEET transporters in pineapple. This information could have important implications for improving fruit crop yield and quality by manipulating SWEET transporter activity.

## Introduction

Sugar is critical for the lives of all living organisms as it provides energy and carbon atoms for metabolic processes. Plants produce their sugar through photosynthesis, but the distribution of sugar from source to sink, sink to cell, and cell to cell requires specific sugar transporters [1]. Multiple sugar transporters control the mobility and availability of sugar content in plants during their growth and development. To date, several families of sugar transporters have been discovered in plants, including sucrose uptake transporters (SUTs/SUCs), sugar transport proteins (STP) and SWEETs (sugars will eventually be exported transporters) [2, 3]. SWEETs are the most recently discovered and functionally diversified sugar transporters. SWEETs, being evolutionarily conserved, have been identified in a wide range of plant genomes, including model species like *Arabidopsis*, as well as economically important crops such as rice, maize, and wheat [4, 5]. Notably, these transporters act as uniporters and exhibit pH-independent activity under *in vivo* conditions [6, 7]. Interestingly, their subcellular localizations have been observed in the plasma membrane [7–9], vacuolar membrane (tonoplast), and Golgi membranes [8, 10, 11]. Such diversification of SWEET transporter localization in eukaryotes or plants differs from prokaryotes. As prokaryotes lack membrane-bound organelles, the diversification of SWEET localization provides a prime example of eukaryotic evolution. Additionally, the evolution of prokaryotic SWEETs (semiSWEETs) into eukaryotic SWEETs is thought to have arisen through gene duplication [12].

To date, significant progress has been made in understanding the transport route and mechanism of SWEET transporters through the use of crystal structures of semiSWEETs, which served as a valuable foundation for the structural studies of SWEETs in eukaryotes [13–15]. Structurally, eukaryotic SWEETs are composed of two triple helix bundle (THB) domains (TM1-TM2-TM3 and TM5-TM6-TM7), each of which is derived from semiSWEETs and separated by a linker helix (TM4). The N-terminal and C-terminal THBs share a similar sequence and are arranged in a parallel orientation [7, 15]. The distinct conformations of the structure allow them to be uniporters to access substrates (sugar molecules) from either the extracellular or cytosolic environments [7, 16, 17]. The substrate-binding pocket is located above the center of the transmembrane region of the transporter protein. Computational analysis has indicated that disaccharides, such as sucrose, are able to easily pass through larger pockets. At the same time, monosaccharides, such as glucose, due to their smaller size, can escape even if the pocket is partially open. However, extracellular residues of the transporter play a significant role in substrate recognition [18, 19].

The evolution of SWEET transporters provides valuable insights into their substrate preferences. Within the plant genome, ~20 paralogs of the SWEET gene family have been identified, and they are commonly categorized into four distinct clades: clades I, II, III, and IV. This classification scheme helps to elucidate the evolutionary relationships and functional diversity among SWEET transporters. Interestingly, the preference for sucrose and hexoses of SWEETs appears to be correlated with phylogeny; for example, SWEETs from clade III mediate sucrose transport. Functional characterization of SWEET transporters in *Arabidopsis* has revealed their crucial roles in various processes, such as seed filling [8], nectar production [20], and pollen nutrition [21, 22].

**Figure 1:**
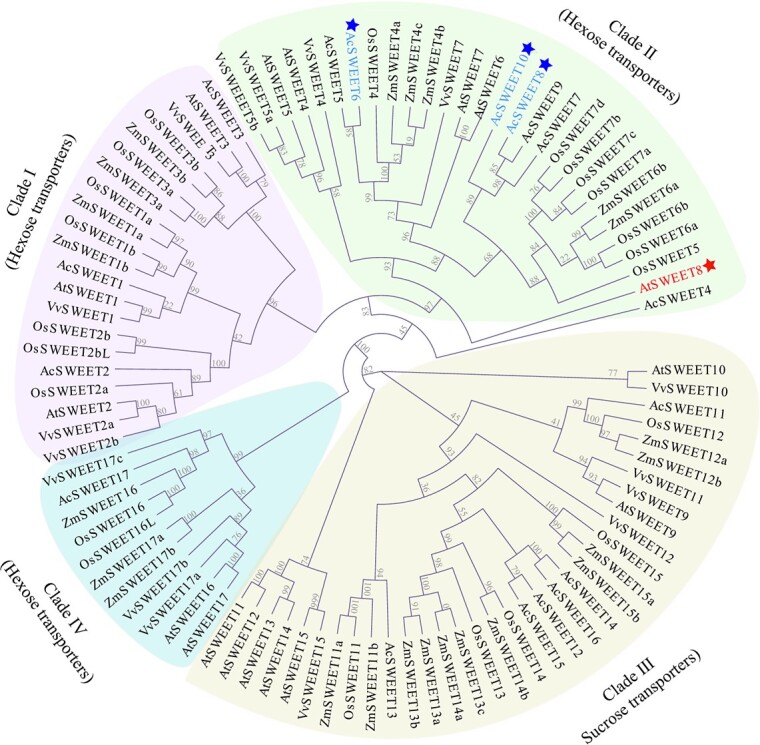
Phylogenetic tree of SWEET proteins from pineapple (Ac), *Arabidopsis* (At), rice (Os), grape (Vv), and maize (Zm). The distances in the tree were calculated from multiple sequence alignment (CLC software) using the maximum likelihood method. Bootstrap values are displayed in percentages. *Arabidopsis* SWEET8 protein is highlighted with red color and pineapple SWEET6/8/10 are represented with blue color.

**Figure 2:**
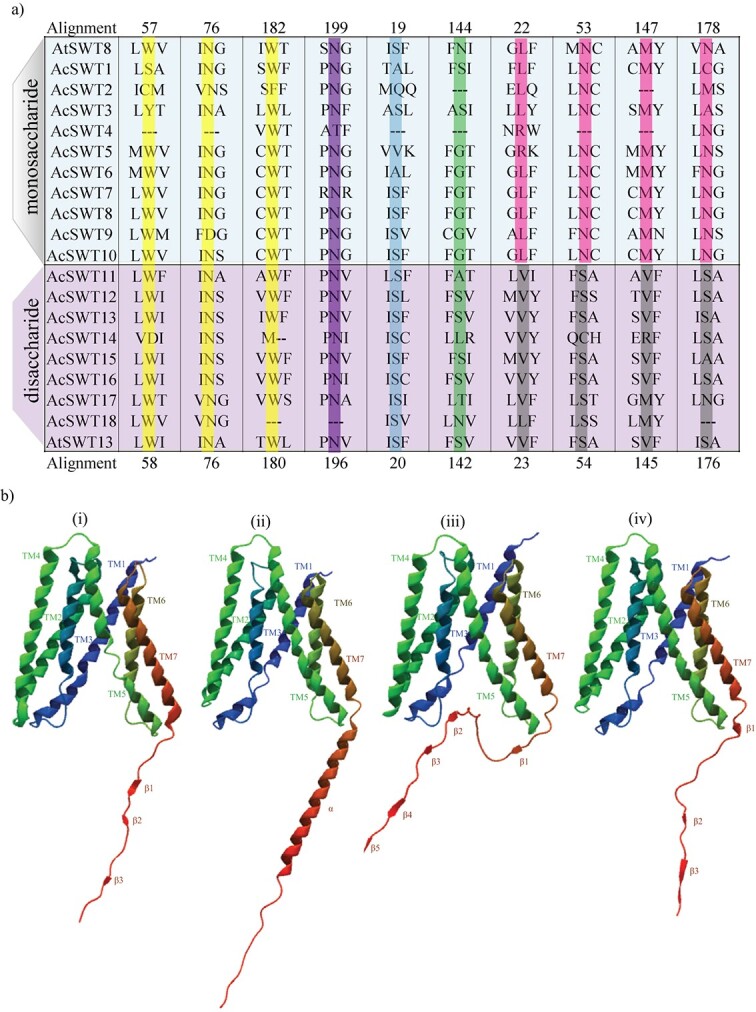
**a** Sequence alignment of the substrate-binding pocket residues in SWEET proteins of pineapple (adapted from the work of Han *et al*. [23]). The alignment includes the protein sequences of AtSWEET8, a glucose transporter, and AtSWEET13, a sucrose transporter, as references. The numbers displayed at the top of the table correspond to the residues of AtSWEET8, while the ones below represent the residues of AtSWEET13. The four conserved residues among di- and monosaccharide-specific SWEETs are highlighted in grey and magenta, respectively. **b** 3D structures of SWEET proteins. In the figure, the numbers i, ii, iii, and iv represent AtSWEET8, AcSWEET6, AcSWEET8, and AcSWEET10 proteins, respectively. The numbers on transmembrane helices (TMs) are represented as TM1 to TM7. TM4 (linker helix) and the C-terminal tails are represented in blue and red colors, respectively.

While significant progress has been made in understanding the function, localization, and substrate specificity of SWEET transporters in *Arabidopsis*, our knowledge of the vastly diverse SWEET transporters in the plant kingdom remains limited. In this study, we aim to expand the knowledge base of SWEET proteins by exploring the available information on SWEETs in pineapple, an economically important plant for which there is currently limited information. Instead of relying solely on linear protein sequences, we employed transmembrane topology and AlphaFold to decipher the structural and physiological roles of SWEETs in pineapple. This study combines 3D structure-based transporter screening with an evolutionary perspective to gain insights into the substrate specificity of SWEET transporters.

## Results

### Three-dimensional structure-based similarity among *Arabidopsis* and pineapple SWEET transporters

We first studied the close relatedness of SWEET transporters by constructing a phylogenetic tree using existing information from rice, grape, maize, *Arabidopsis*, and pineapple. Most pineapple SWEETs were found to be closely associated with their corresponding SWEETs from *Arabidopsis*, forming clades I, II, III, and IV (Fig. 1). The average sequence identity of pineapple SWEETs was found to be 50%. Notably, the C-termini of the pineapple SWEET proteins displayed less conservation and exhibited varying sequence lengths (Supplementary Data Fig. S1). To determine their potential target sugar for transport, the pineapple SWEETs were compared with well-characterized glucose (OsSWEET2b, AtSWEET8) and sucrose (AtSWEET13) transporters. The results revealed sequence similarities between pineapple SWEETs and known glucose and sucrose transporters, with AcSWEET1, 2, and 3 having a high sequence similarity to OsSWEET2b (clade I). At the same time, AcSWEET5, 6, 7, 8, 9, and 10 showed similarities to AtSWEET8 (clade II). AcSWEET11, 12, 13, 14, and 15 shared similarities with AtSWEET13 (clade III), while AcSWEET16 and 17 had similarities to AtSWEET13 (clade III). AcSWEET4 and 18 displayed lesser sequence similarities with AtSWEET8 and AtSWEET13 sequences (Figs 1 and2a and Supplementary Data Table S1).

We then selected three pineapple SWEET proteins (AcSWEET6, 8, and 10) from clade II for comprehensive structural and functional characterization. Our choice was based on their similarity to the well-characterized AtSWEET8 protein. Moreover, the selection of these three pineapple SWEET proteins was also due to their phylogenetic relationship, as well as their similarity in physiochemical properties and predicted subcellular localization (Supplementary Data Table S2) in relation to AtSWEET8. Given the comparable nature of pineapple SWEET6, 8, and 10 to AtSWEET8, we proposed that they might facilitate glucose transport in a manner similar to the SWEET8 protein in *Arabidopsis*. This hypothesis is supported by the conserved functions observed in SWEET proteins across various plant species.

### Screening of glucose transporter among AcSWEETs via homology-based search

The observed similarities between AtSWEET8 and AcSWEET10, as well as the differences from AcSWEET6 and 8, indicate that AcSWEET10 has closer resemblance to AtSWEET8 and may exhibit a greater affinity for glucose transport compared with AcSWEET6 and 8 (Supplementary Data Fig. S2). An analysis of the 3D structures of selected pineapple SWEET transporters in reference to the AtSWEET8 transporter could enable the prediction of the substrates transported by them and provide insights into their role in facilitating sugar transport within the plant. Therefore, to determine whether AcSWEET6, 8, and 10 would transport glucose, the 3D structures of AtSWEET8, AcSWEET6, 8, and 10 were predicted using the AlphaFold server. The structures indicated that the proteins have a common structural arrangement with seven transmembrane segments organized into two units, connected by an inversion linker, TM4. The structural arrangements of AcSWEET6, 8, and 10 were in line with previous findings in rice and *Arabidopsis* (Fig. 2b). Despite the similarities, significant variations were observed in the C-terminal tails of AcSWEET6 and 8. For instance, AcSWEET8 had two additional β-sheets compared with AtSWEET8, while AcSWEET6 had an α-helix instead of β-sheets. In contrast, AcSWEET10 was structurally similar to AtSWEET8 (Fig. 2b). These variations in the 3D structures of AcSWEET6 and AcSWEET8 could play a role in determining substrate specificity and transport activity and may affect the glucose transport activity of AcSWEET6 and AcSWEET8. Altogether, the results of substrate specificity and comparative structural analysis indicate that only AcSWEET10 shares a close structural similarity with AtSWEET8, suggesting that it has the highest affinity for glucose transport among AcSWEET6, 8, and 10.

### AcSWEET6, AcSWEET8, and AcSWEET10 localize to the plasma membrane

**Figure 3:**
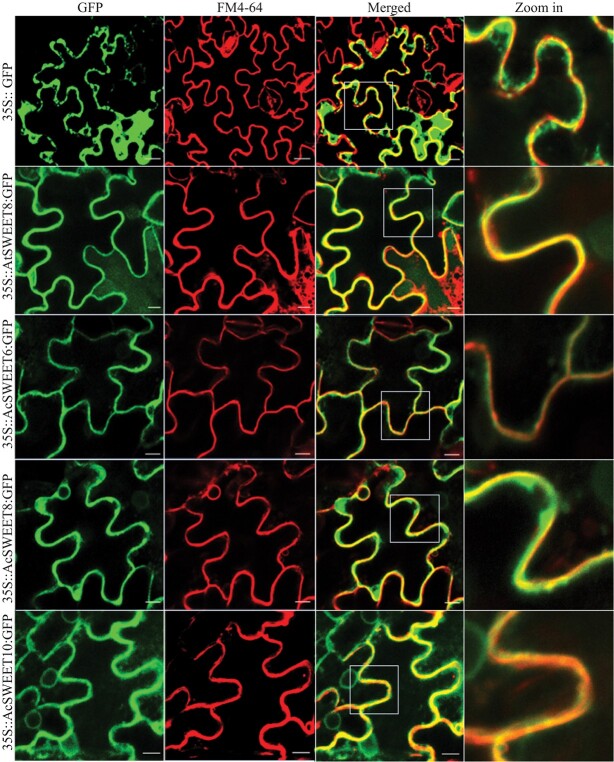
Pineapple SWEET proteins are localized to the plasma membrane. Subcellular localization of AcSWEET6, 8, and 10 proteins. The 35S::GFP, 35S::AtSWEET8:GFP, 35S::AcSWEET6:GFP, 35S::AcSWEET8:GFP, and 35S::AcSWEET10:GFP fusion proteins were transiently expressed in tobacco epidermal cells. After 48 h incubation, leaf segments were incubated with FM4-64 for 10 min and the abaxial epidermal cells were visualized by fluorescence microscopy. Scale bars: 25 μm.

The substrate specificity and comparative structural analysis indicated that AcSWEET6, 8, and 10 could transport glucose, so we checked their subcellular localization to confirm the putative functional location. The subcellular localizations of AcSWEET6, 8, and 10 were identified using AcSWEET6, 8, and 10 GFP fusion proteins in tobacco epidermal cells (Fig. 3). The GFP signals were compared with the earlier reported plasma membrane-localized AtSWEET8-GFP [7, 22] and the GFP control vector (35S::GFP), which was distributed throughout the cells. The fluorescence signals for AcSWEET6, 8, and 10 were observed in the plasma membrane (Fig. 3). The localization results also indicated that the SWEETs show conservation for subcellular localization.

### Expression of *AcSWEET* genes during pineapple pollen development

**Figure 4:**
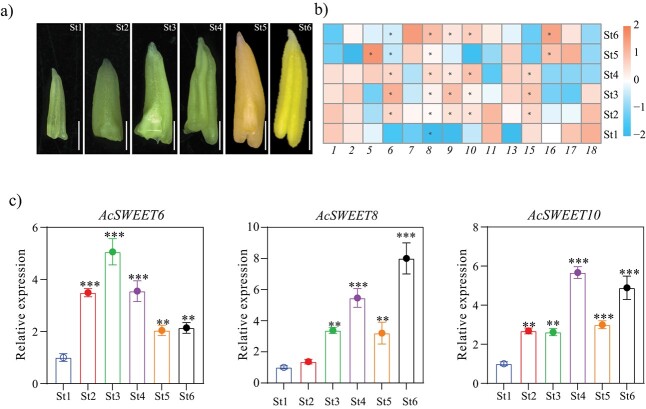
Pineapple *SWEETs* show differential expression in the anthers. **a** Morphological characteristics of the pineapple stamen tissues used for RNA-seq analysis were harvested at six stages (St1–St6). Scale bar: 1 mm. **b** Heat map of expression profiles of *AcSWEET*s in stamen tissues at different stages. The numbers below represent the pineapple *SWEET* genes. The heat map was created based on the log_2_ (FKPM +0.01) value of *AcSWEET*s and normalized by row. FKPM values >30 are marked with an asterisk. Differences in gene expression changes are shown in color, as shown in the scale to the right. **c** Relative expression levels of *AcSWEET6*, *8*, and *10* genes from six developmental stages of stamens. Vertical bars represent the mean ± standard error of three biological replicate assays. Asterisks denote statistical significance compared with stage 1 as judged by Student’s *t*-test (^**^P < .01, ^***^P < .001).

The plasma membrane-specific cellular localization of AcSWEET6, 8, and 10 resembled that of the well-characterized AtSWEET8. Subsequently, we examined the organ-specific expression of AtSWEET8, which was observed in the stamen (Supplementary Data Fig. S3). Nevertheless, the localization of *AcSWEET*s during developmental stages of stamens remained unknown. To address this gap, we analyzed the expression of *AcSWEET*s using RNA-seq data from six developmental stages (St1–St6) of the stamen (Fig. 4a and b). The results showed that 66% of the *AcSWEET* genes were upregulated during all the developmental stages of stamens. Among them, *AcSWEET6*, *AcSWEET8*, and *AcSWEET10* expressions were detected during pineapple stamen development. The expression patterns of *AcSWEET6*, *8*, and *10* were further validated using qRT–PCR in the developing stamen (Fig. 4c). Altogether, these results suggest that pineapple SWEET transporters are similar to AtSWEET8 not only in terms of 3D structure and cellular localization but also of tissue-specific expression.

### Pineapple SWEET10 transports glucose

**Figure 5:**
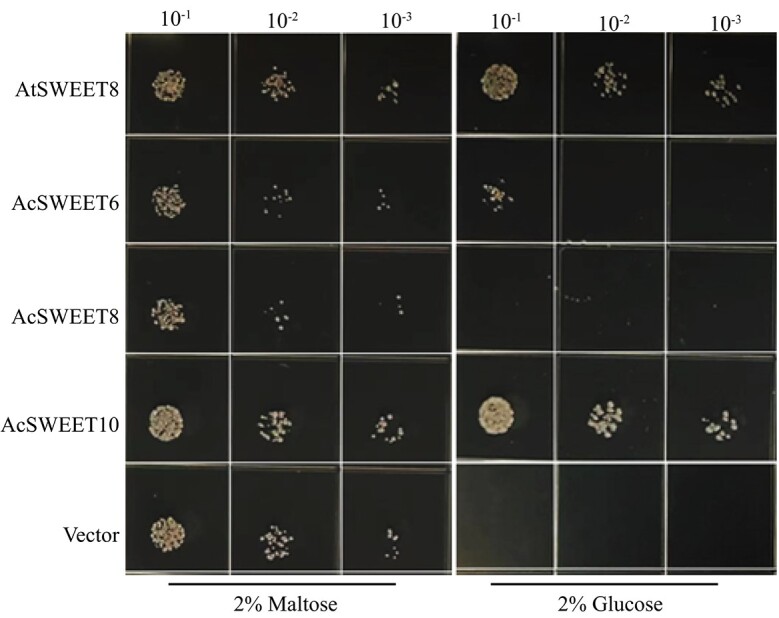
AcSWEET10 transports glucose in yeast. Glucose complementation assay of AcSWEET6, AcSWEET8, and AcSWEET10, where AtSWEET8 (positive control) and vector (without insert, as negative control) were expressed in the hexose transport-deficient yeast mutant EBY.VW4000. The transformed yeast colonies were diluted 10-fold and cultured on synthetic deficient medium without tryptophan (SD/−Trp) supplemented with 2% (w/v) maltose (as growth control) and 2% (w/v) glucose. Images were taken after culture plates had been incubated at 30°C for 3–5 days.

Although the homology-based identical pineapple SWEET transporters have comparable cellular localization and tissue-specific expression, the question remained whether pineapple SWEET transporters are capable of glucose transport. Due to technical difficulties with pineapple genetic modification, it is almost impossible to knock out each glucose transporter in the plant. As an alternative and the most reliable approach, we took advantage of the hexose transport-deficient yeast mutant EBY.VW4000 and performed the glucose transport assay *in vivo*. To achieve this, expression vectors harboring the coding sequences of *AcSWEET6*, *AcSWEET8*, and *AcSWEET10* were transformed into EBY.VW4000. Transformants were allowed to grow in synthetic deficient (SD/−Trp) medium supplemented with maltose (as growth control) and glucose. The yeast complementation assay showed that AcSWEET10 complements the mutant strain and grows well on glucose-supplemented medium similarly to AtSWEET8, while AcSWEET6 grows weakly. However, the yeast carrying AcSWEET8 and the vector (without insert) did not show detectable growth on medium rich in glucose (Fig. 5). These heterologous transport assays highlight that AcSWEET10 has a maximum affinity to transport glucose compared with AcSWEET6 and 8.

**Figure 6:**
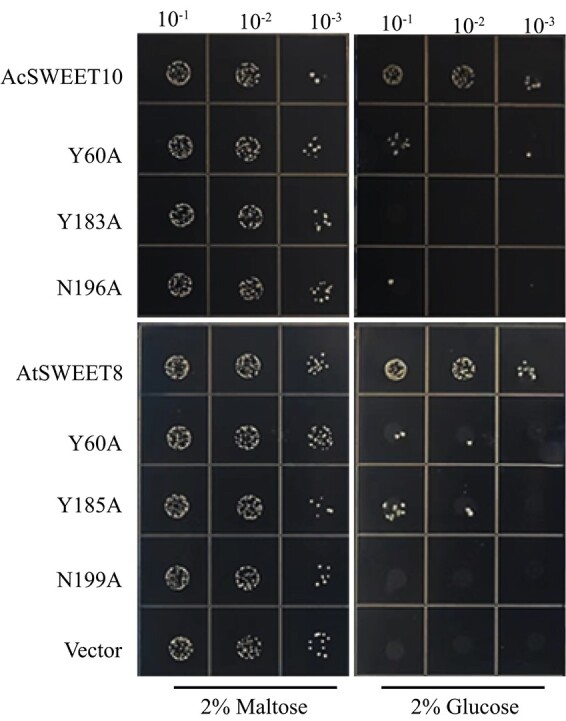
Conserved amino acids of AcSWEET10 play a crucial role in glucose transport. A glucose transport assay of the indicated strains and vector (without insert, as negative control) was carried out in the hexose transport-deficient yeast mutant EBY.VW4000. The transformed yeast colonies were diluted 10-fold and cultured on synthetic deficient medium without tryptophan (SD/−Trp) supplemented with 2% (w/v) maltose (as growth control) and 2% (w/v) glucose. Images were taken after culture plates had been incubated at 30°C for 3–5 days.

To investigate the glucose transport ability of AcSWEET10 in more detail, we conducted site-directed mutagenesis experiments targeting conserved amino acids. Specifically, we focused on the predicted significance of Y60, Y183, and N196 residues in AcSWEET10, as well as Y60, Y185, and N199 residues in AtSWEET8 (Supplementary Data Fig. S5). These amino acids were substituted with alanine residues, following the conservation pattern observed in OsSWEET2b, a well-known transporter with established glucose transport capabilities [18]. To evaluate the impact of these mutations, we expressed the mutated versions of both AtSWEET8 and AcSWEET10 in the EBY.VW4000 yeast strain. As anticipated, yeast cells expressing the mutated proteins of AtSWEET8 and AcSWEET10 exhibited normal growth on maltose-supplemented media. However, when cultivated on glucose-supplemented plates, the yeast cells expressing the mutated proteins displayed significantly reduced growth, indicating a defect in glucose transport (Fig. 6). These findings highlight the critical role of the substituted amino acids, Y60, Y183, and N196, in facilitating glucose transport. Moreover, they suggest that these specific amino acids are likely involved in recognizing and transporting glucose across the pineapple cell membrane.

### AcSWEET10 functions as a glucose transporter in *Arabidopsis*

**Figure 7:**
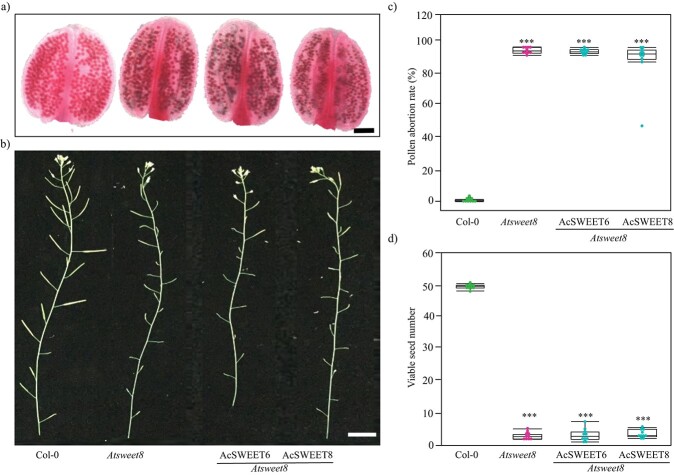
AcSWEET6 and 8 do not complement *Arabidopsis sweet8* function. **a** Alexander dye-stained anthers showing non-viable pollen in non-complemented plants, similar to the mutant. Scale bar: 50 μm. **b** Primary inflorescence stems showing shorter siliques of non-complemented plants. Scale bar: 3 cm. **c** Graph showing pollen abortion rate. **d** Graph showing viable seed number. Asterisks above the columns indicate significant differences compared with Col-0. ^*^P < .001.

To investigate whether the AcSWEET10 of pineapple has a similar role in anther development, ectopic expression of AcSWEET6, 8, and 10 in the *Atsweet8* mutant was studied to determine their complementation ability. In agreement with our substrate specificity and comparative structural analysis results, AcSWEET6 and 8 failed to complement the mutant phenotype. This was evident from pollen viability analysis using Alexander staining, which showed that most of the pollen in these lines was non-viable (Fig. 7a and b). The percentages of pollen abortion and viable seed numbers in these lines were similar to those in the mutant (Fig. 7c and d), indicating that AcSWEET6 and 8 do not transport glucose and therefore failed to complement the *Atsweet8* mutant.

**Figure 8:**
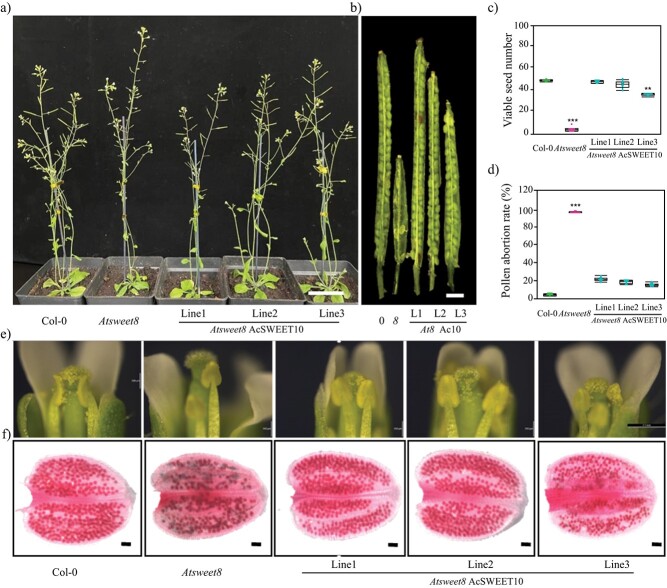
AcSWEET10 functions as a glucose transporter and complements *Arabidopsis sweet8* mutant. **a** Five-week-old plants showing silique growth from the initial stages. Scale bar: 3 cm. **b** Representative photographs showing variable extents of restored silique lengths in three complemented lines (L1, L2, and L3) with reference to *Atsweet8* mutant. Scale bar: 1 mm. **c** Graph representing viable seed number per silique (harvested from position 5 onwards from the main inflorescence stem). **d** Graph showing pollen abortion rate. **e** Brightfield microscopy of flowers showing pollen on anthers and stigma of Col-0, *Atsweet8*, and three complemented lines, the flowers showing abundant pollen on stigma of complemented lines. Scale bar: 20 μm. **f** Viable pollen stained bright red was observed in the Alexander-stained anthers. Scale bar: 200 μm. Values represent means + standard error (*n* = 10 siliques per genotype) and asterisks above the columns (in **c** and **d**) indicate significant differences compared with Col-0. ^**^P < .01, ^***^P < .001.

In contrast, the ectopic expression of AcSWEET10 was able to restore the defective pollen phenotype of the *Atsweet8* mutant. AcSWEET10 expression fully rescued the low-fertility phenotype of the mutant plants. The complemented lines displayed a significant improvement in silique development and seed set (Fig. 8a and b), with the viable seed number nearly identical to that of the wild type (Fig. 8c) and a significant reduction in the pollen abortion rate in the complemented lines (Fig. 8d). The anthers showed viable pollen in complemented lines, similar to the wild type (Fig. 8e). Alexander staining showed that most of the pollen in the AcSWEET10 lines was viable, resulting in a restoration of the mutant phenotype (Fig. 8f).

**Figure 9:**
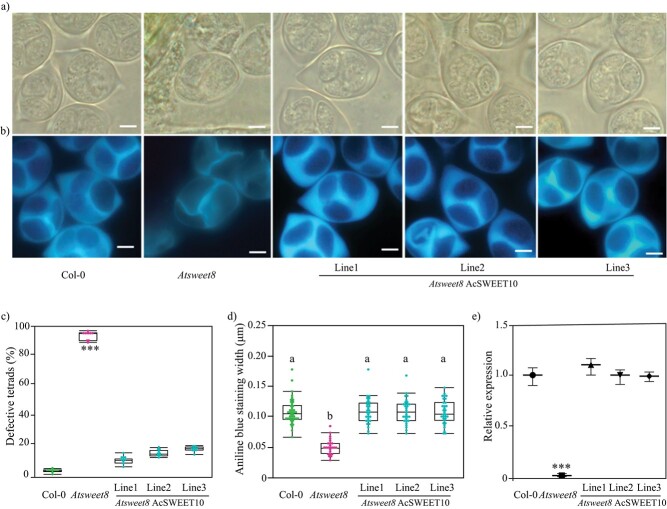
AcSWEET10 regulates callose 5 synthase levels and maintains tetrads during microspore development. **a** DIC images showing wall architecture of tetrads of wild type (Col-0), *Atsweet8*, and three AcSWEET10 + *Atsweet8* complemented lines. **b** Cytochemical staining with aniline blue of tetrads of in Col-0, *Atsweet8*, and three complemented lines Scale bar: 20 μm. **c** Percentage of defective tetrads in Col-0, *Atsweet8*, and three complemented lines. **d** Aniline blue staining width quantified for Col-0, *Atsweet8*, and one complemented line. The statistical test was based on Tukey’s honest test. Groups labeled with the same letter are not statistically different from each other (α = 0.05). **e** Relative expression levels of *callose 5 synthase* in anthers (harvested at tetrad stage) from Col-0, *Atsweet8*, and three complemented lines. Values represent means ± standard error of three biological replicates. ^***^P < .001.

In a previous study, mutation of *Atsweet8* significantly reduced the expression of *CalS5* (key enzyme for callose biosynthesis), resulting in the thinning of the callose wall of the *Atsweet8* microspore [22, 24]. To investigate whether fertility restoration by AcSWEET10 is associated with callose deposition, we checked the deposition of callose and *CalS5* expression in the wild-type, mutant, and complemented lines. DIC microscopy showed an improved callose wall around developing microspores at the tetrad stage in complemented lines compared with thinner, degenerated walls around mutant microspores (Fig. 9a).

Callose fluorescence was comparable in wild-type and complemented lines in contrast to *Atsweet8* (Fig. 9b and d). The percentage of defective tetrads was significantly reduced to up to 20% in complemented lines (Fig. 9c). The relative expression of *CalS5* in complemented lines was similar to that of wild type, in contrast to the mutant (Fig. 9e). Taken together, these results suggest that the abnormal callose deposition related to the reduced fertility in the *Atsweet8* mutant is complemented by AcSWEET10, indicating that AcSWEET10 has a similar function to SWEET8 in pineapple.

## Discussion

The SWEET gene family is recognized for its pivotal role in the facilitation of sugar transport across cellular membranes, a function critical for various physiological and developmental processes in plants [5, 21, 25–30]. Extensive investigations into the SWEET family in model plants such as *Arabidopsis* and rice have yielded valuable insights into their structural and functional characteristics. In *Arabidopsis*, the 17-member SWEET family is categorized into four clades, each associated with distinct sugar transport functions, contributing to our understanding of sugar allocation and homeostasis within plants [23]. In general, clade III SWEET proteins tend to transport sucrose, whereas clades I, II, and IV exhibit a preference for hexose transport [31]. Drawing inspiration from this knowledge base, our study sought to extrapolate these insights to the pineapple (*Ananas comosus*) SWEET gene family, specifically aiming to predict and validate putative glucose transporters within this tropical fruit crop. While *Arabidopsis* and pineapple represent divergent botanical lineages, we leveraged the conserved principles of protein structure and function to bridge this evolutionary gap. Through a comprehensive approach that integrated structural analysis and functional assays, we elucidated potential glucose transporters among the pineapple SWEET proteins, thereby contributing novel perspectives to the broader understanding of sugar transport mechanisms in plants. Finally, we validated these predictions by exploring the glucose transport activity of AcSWEET6, 8, and 10 in a yeast transport assay and functionally complementing the *Arabidopsis sweet8* mutant.

Previous studies on SWEET structures have provided important information about their putative function in various plants [7, 22, 32]. SWEETs have a single sugar-binding site within the protein and undergo conformational changes to transport the sugar [19, 33]. By analyzing homologous amino acid sequences and the amino acid configuration of the binding pocket, we identified a set of conserved residues that were indicative of glucose or sucrose transporters (Fig. 2a and Supplementary Data Fig. S1). Based on these conserved residues, we predicted five sucrose transporters (AcSWEET11, 12, 13, 15, and 16) and nine glucose transporters (AcSWEET1, 2, 3, 5, 6, 7, 8, 9, and 10) in pineapple (Fig. 2a).

*Arabidopsis* SWEET proteins play critical roles in the transport of glucose directly or in the form of sucrose between compartments, cells, and organs [7, 24, 31]. AtSWEET8 is a well-known plasma membrane glucose transporter, and its loss of function leads to severely defective pollen and a reduction of up to 90% in seed set [22]; therefore, it was utilized as the reference for this study. Clade III members (AcSWEET6, 8 and 10) displayed high similarity in their physicochemical properties to AtSWEET8 and localize to the plasma membrane (Fig. 3). The glucose molecule follows a specific path by interacting with residues across the SWEET8 cavity [18]. Notably, the same residues for glucose entry and exit in the AcSWEET6, 8 and 10 structures indicated their potential for glucose transport activity (Supplementary Data Fig. S1). Moreover, similar to *AtSWEET8*, the high expression of *AcSWEET6*, *8*, and *10* in pineapple stamens showed their potential role in sugar supply in male reproductive organs (Fig. 4a and c and Supplementary Data Fig. S2).

Experimental verification of the putative transporters using multiple hexose transporter-deficient yeast showed that only AcSWEET10 could enable robust growth on glucose (Figs 5 and6). Notably, AcSWEET10 might have retained a conserved structure that facilitates glucose transport, as evidenced by both its preserved functional characteristics and its ability to rescue the growth of the *Atsweet8* mutant, which has impaired sugar transport (Figs 2b and8). Interestingly, the contrasting behavior of AcSWEET6 and AcSWEET8 is intriguing. Despite sharing identical conserved residues, both proteins failed to transport glucose efficiently and were unable to complement the sugar transport deficiency in the mutation. This disparity underscores the critical role of the C-terminal region in determining their functionality (Fig. 7).

Previous reports on rice SWEET2b have revealed the functional significance of specific residues in glucose transport. One such residue, Y61 of TM2, is considered crucial as its mutation disrupts the extra-facial gate of the transporter, thereby impeding the conformational transitions necessary for glucose movement. Further studies by Selvam *et al*. [18] revealed that Y184 of TM7 in OsSWEET2b, located at the extracellular side, establishes polar and hydrophobic interactions with glucose, thereby facilitating glucose transport towards the center of the transporter. Moreover, they demonstrated that N190 of TM7 in OsSWEET2b is involved in strong polar interactions with other residues, such as Y61 of TM2, enabling the closure of the transporter in an occluded conformation [15, 18]. We investigated the roles of conserved residues in AcSWEET10 by conducting site-directed mutagenesis experiments. These experiments targeted residues corresponding to key residues in OsSWEET2b that are crucial for glucose recognition and transport [13, 34]. Specifically, we conducted site-directed mutagenesis on Y60 of TM2 and Y183 of TM7 of AcSWEET10, which align with Y61 of TM2 and Y184 of TM7 of OsSWEET2b, respectively. The results of the mutagenesis experiments on AcSWEET10 were consistent with predictions from the computational analysis, validating the importance of these residues in glucose recognition and transport. Our experimental findings clearly demonstrated that these substitutions compromised the glucose transport activity of both AcSWEET10 and AtSWEET8 (Fig. 6 and Supplementary Data Fig. S5). These findings provide additional evidence supporting the key roles of these conserved residues in recognizing and delivering glucose molecules. Overall, in combination with computational analyses and previous research, our results underscore the significance of the conserved residues, such as Y60 of TM2, Y183 of TM7, and N196 of TM7, in AcSWEET10, in facilitating glucose transport.

The 3D structure of AcSWEET6, 8, and 10 proteins using the AlphaFold algorithm showed high similarity overall, but they differed significantly at the C-terminal region (Fig. 2b). The predicted structures suggest that the C-terminal region of AcSWEET6 forms an extra α-helix, which is absent in AcSWEET8 and 10. In addition, the C-terminal region of AcSWEET8 possessed an additional β-sheet which changed the structure leading to hindrance of the pore (Fig. 2b). Previously, it was shown that three mutant alleles of the *Atsweet8* gene (Salk_142803, Salk_062567, and Salk_092239) had a T-DNA insertion in the first exon, fifth exon, and first intron, respectively (Supplementary Data Fig. S4). However, the stronger defect in phenotype pollen of the *AtSWEET8* gene was observed in the *rpg1* mutant, a T-DNA insertion in the last intron. Consequently, in RT–PCR of the *Atsweet8* mutant, the T-DNA insertion primarily affected the transcription of the last exon, which corresponds to the C-terminal tail of its protein [22]. Altogether this suggests that the C-terminal region of SWEET8 plays an essential role in its function. Besides, several studies indicate the significance of C-terminal regions for the functions of different transporters [35–37]. The structural difference of the AcSWEET6 and 8 at the C-terminal compared with AcSWEET10 and AtSWEET8 could impact the glucose transport activity of the proteins AcSWEET6 and 8. Consistently, the present study revealed that AcSWEET10 has a structure similar to AtSWEET8 and displays higher glucose transport activity than SWEET6 and 8 (Figs 2b and5), most likely due to the difference in their C-terminal region, which may be hindering sugar transport.

**Figure 10:**
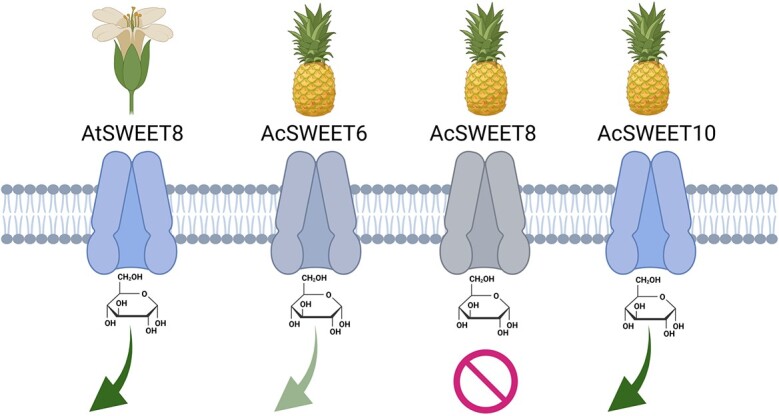
A schematic model representing the glucose transport mechanism in SWEET proteins. The figure shows the inward open conformations of SWEET proteins, including AtSWEET8, AcSWEET6, AcSWEET8, and AcSWEET10. The transport process is symbolized by arrows, color-coded to indicate varying levels of transport activity. Dark green arrows signify robust glucose transport, light green arrows denote weaker transport, and a `stop' sign is employed to indicate restricted glucose transport. Notably, the direction of the arrows also signifies the exit of glucose molecules through the transporter's channel.

To summarize, this research has highlighted the significance of the C-terminal region for the appropriate functioning of SWEETs. The results of the study revealed that AcSWEET10 has a conserved function and imitates the overall structure of AtSWEET8, implying that residues in the C-terminal region can have an impact on transporter activity (Fig. 10 and Supplementary Data Fig. S6).

Further investigation is needed to explore the C-terminal regions of SWEET transporters and the possible presence of motifs interacting with glucose, particularly in the case of AcSWEET6 and 8. Overall, these findings have important implications for understanding plant physiology and metabolism and developing strategies to improve crop yield and quality. Further investigation in this area could develop novel tools for manipulating sugar transport in plants, which could have far-reaching benefits for agriculture and food security.

## Materials and methods

### Bioinformatic analysis

The pineapple SWEET proteins sequences identified by Guo *et al*. [50] were obtained from Phytozome (https://phytozome.jgi.doe.gov/pz/portal.html). Protein sequences of *Arabidopsis*, rice (*Oryza sativa*), and maize were downloaded from TAIR (http://www.arabidopsis.org), the Rice Data Center of China (http://www.ricedata.cn/gene/index.htm), and MaizeGDB (https://www.maizegdb.org/), respectively. The ExPASy (http://ca.expasy.org/prosite/) proteomics server was used for the physicochemical properties. The WoLF PSORT (http://wolfpsort.hgc.jp) algorithm was utilized to predict the subcellular localization and TMHMM Server v.2.0 (http://www.cbs.dtu.dk/services/TMHMM/) was used to predict transmembrane helical domains available. The protein sequences were aligned, and the phylogenetic tree was constructed using MEGA (version 11.0) with the maximum likelihood method [38]. The tree was built and assessed with a bootstrap test with 1000 iterations [39]. The transmembrane topologies were predicted and generated with Protter [40].

### Homology-based modeling

The structure models of pineapple SWEET proteins in inward-facing states were built from AlphaFold [41] and were visualized and analyzed using the CLC genomics workbench (version 22.0.2). The models were compared by using available structural templates: AtSWEET8 and AtSWEET13.

### Plant materials

*Arabidopsis* mutant (*Atsweet8*) was grown in a growth chamber in potted soil under a 16-h light/8-h dark regime at 22 ± 2°C. Transformation of *Arabidopsis* plants was done with *Agrobacterium* strain GV3101 using the floral dip method [42]. Pineapple plants (MD2 variety) were acclimated in soil mix [peat moss:perlite = 2:1 (v/v)] in plastic pots in a walk-in growth chamber. The growth chamber was maintained at 25 ± 2°C with a 16-h light/8-h dark photoperiod and 70% humidity, as reported earlier [43]. The pineapple anthers were collected and photographed at six developmental stages as described before [44], quickly frozen in liquid nitrogen, and stored at −80°C until RNA extraction.

### Total RNA isolation and quantitative-real time PCR analysis

Total RNA was isolated using the RNeasy kit (Qiagen, MD, USA) followed by treatment with DNaseI (Thermo Fisher Scientific, CA, USA), and was reverse-transcribed using the ThermoScript RT–PCR kit (Thermo Fisher Scientific, CA, USA). The qRT–PCR reactions were set up with FastStart DNA Master SYBR Green I master mix (Takara, Japan). For each analysis, two technical replicates from three biological replicates were taken, and pineapple and *Arabidopsis EF1α* genes were used to normalize the mRNA levels. Finally, the fold change of genes was calculated using the 2^−∆∆CT^ method [45]. Primers used in this study are listed in Table S3.

### RNA-seq analysis

Transcriptome data of pineapple stamen developmental stages were used to investigate the expression level of SWEET genes [44]. Briefly, the sequencing reads were aligned to the pineapple genome using TopHat v2.1.1 with default parameters. Transcript abundance was calculated as fragments per kilobase of exon model per million mapped reads (FPKM). The heat map was generated using pheatmap R software based on log_2_ (FPKM + 0.01).

### Transient expression of pineapple SWEETs in tobacco epidermal cells

Agrobacterial cultures of *35S::AcSWEET6:GFP*, *35S::AcSWEET8:GFP*, *35S::AcSWEET10:GFP*, *35S::GFP* (empty vector), and *35S::AtSWEET8:GFP* (as positive control) fusion constructs were pelleted and resuspended in the infiltration media [46]. Using a needleless syringe, the suspension (OD 0.5) was infiltrated into the abaxial surface of fully expanded tobacco leaves of 4-week-old plants. GFP signals were detected at different time intervals between 48–72 h post-infiltration (hpi) with a TCS SP8 microscope (Leica).

### Pollen viability analysis

Anthers were dissected from flower buds at stage 12 (before anthesis) and fixed in Carnoy fixative (absolute ethanol:chloroform:acetic acid, 6:3:1) for at least 2 h. Afterwards, anthers were stained in Alexander stain [47]. For aniline staining, anthers at the tetrad stage were gently fixed overnight in FAA. The anthers were then stained in 0.1% (w/v) aniline blue in 0.1 M sodium phosphate (pH 9.0) and incubated in the dark for 1–2 h. Stained anthers were mounted on 30% glycerol and viewed with UV epifluorescence (365 nm excitation and a 420 nm long-pass emission filter).

### Functional complementation of AcSWEETs in *Arabidopsis* (*sweet8*) mutant

PCR fragments of the complete *AcSWEET6*, *AcSWEET8*, and *AcSWEET10* coding sequences were amplified from flower-specific cDNA using specific primers. The fragments were cloned into the entry vector pENTR™/D-TOPO^®^ and then subcloned into the Gateway destination vector pGWB505 [48] using LR Clonase II (Invitrogen). After confirmation by sequencing, the constructs were transformed into *Agrobacterium* and finally into homozygous *Atsweet8/rpg1 Arabidopsis* plants using the floral dip method. The transgenic plants were selected on media plates containing 50 mg L^−1^ hygromycin.

### Glucose transport assay in yeast

For the yeast transport assays, the *Saccharomyces cerevisiae* strain EBY.VW4000 [49] was used, as described previously [28]. The ORFs of *AcSWEET6*, *AcSWEET8*, and *AcSWEET10* were amplified using specific primer combinations. To investigate the impact of specific mutations on the glucose transport ability of AcSWEET10, site-directed mutagenesis was performed at three different positions (Y60A, Y183A, and N196A). As a reference for the site-directed mutagenesis experiment, *Arabidopsis* SWEET8 was also mutated at three similar amino acids (Y60A, Y185A, and N199A), which have been previously proposed to be responsible for glucose transport [18]. Mutagenic primers were designed and PCR-based site-directed mutagenesis was carried out using high-fidelity DNA polymerase. The amplified fragments were then infused into the modified yeast expression vector pGBKT7 and sequenced to verify the correctness of the clones. The expression clones and an empty vector (without insert) were transformed into the yeast strain EBY.VW4000. Yeast transformants were then selected on a synthetic deficient medium without tryptophan (SD/−Trp) supplemented with 2% (w/v) maltose as a carbon source. Transformed yeast cells were grown in SD/−Trp liquid medium with 2% (w/v) maltose and were incubated overnight at 30°C until the optical density at 600 nm (OD_600_) reached 0.5. The adjusted OD_600_ (~0.2) with water was serially diluted (×10, ×100, and ×1000) and spotted onto SD/−Trp solid medium with 2% (w/v) maltose (control) and 2% (w/v) glucose. All transformants were incubated at 30°C, and growth was documented after 3 days in maltose media and 3–5 days in glucose media.

## Acknowledgements

We especially thank Dr Binghua Wu (Fujian Agriculture and Forestry University, China) for kindly providing the yeast mutant strain EBY.VW4000 and Prof. Zhong-Nan Yang (Shanghai Normal University, China) for sharing *Atsweet8* seeds. This work was supported by the Science and Technology Major Project of Guangxi (Gui Ke AA22068096), the Science and Technology Innovation Project of Pingtan Science and Technology Research Institute (PT2021007, PT2021003), the General Project of Fujian Natural Science Foundation (2020 J01594), the Project of Guangxi Featured Fruit Innovation Team on Pineapple Breeding and Cultivation Post under the National Modern Agricultural Industry Technology System (nycytxgxcxtd-17-05), and the Guangxi Academy of Agricultural Sciences Basic Research Project (Gui Nong Ke 2021YT046). The funding bodies played no role in the design of the study and collection, analysis and interpretation of the data, and writing the manuscript.

## Author contributions

M.A. and Y. Q. designed the study. B.F. performed all the experiments except RNA-seq. L.W., X.W., and P.Z. performed RNA-seq. B.F, M.A.A., M.A., and Y.Q. analyzed the data and wrote the paper. All authors have read and approved the manuscript.

## Data availability

The datasets analyzed in this study are publicly available with the accession numbers PRJNA331052, PRJNA305042, and PRJEB38680.

## Conflict of interest

The authors declare that the research was conducted in the absence of any commercial or financial relationships that could be construed as a potential conflict of interest.

## Supplementary data

Supplementary data is available at *Horticulture Research* online.

## Supplementary Material

Web_Material_uhad175

## References

[1] Hedrich R , Sauer N, Neuhaus HE. Sugar transport across the plant vacuolar membrane: nature and regulation of carrier proteins. Curr Opin Plant Biol. 2015;25:63–70 26000864 10.1016/j.pbi.2015.04.008

[2] Johnson DA , Thomas MA. The monosaccharide transporter gene family in *Arabidopsis* and rice: a history of duplications, adaptive evolution, and functional divergence. Mol Biol Evol. 2007;24:2412–23 17827171 10.1093/molbev/msm184

[3] Büttner M , Sauer N. Monosaccharide transporters in plants: structure, function and physiology. Biochim Biophys Acta. 2000;1465:263–74 10748259 10.1016/s0005-2736(00)00143-7

[4] Li X , Si W, Qin Q et al. Deciphering evolutionary dynamics of SWEET genes in diverse plant lineages. Sci Rep. 2018;8:13440 30194417 10.1038/s41598-018-31589-x PMC6128921

[5] Xue X , Wang J, Shukla D et al. When SWEETs turn tweens: updates and perspectives. Annu Rev Plant Biol. 2022;73:379–403 34910586 10.1146/annurev-arplant-070621-093907

[6] Chen L-Q , Qu XQ, Hou BH et al. Sucrose efflux mediated by SWEET proteins as a key step for phloem transport. Science. 2012;335:207–11 22157085 10.1126/science.1213351

[7] Chen LQ , Hou BH, Lalonde S et al. Sugar transporters for intercellular exchange and nutrition of pathogens. Nature. 2010;468:527–32 21107422 10.1038/nature09606 PMC3000469

[8] Chen L-Q , Lin IW, Qu XQ et al. A cascade of sequentially expressed sucrose transporters in the seed coat and endosperm provides nutrition for the *Arabidopsis* embryo. Plant Cell. 2015;27:607–19 25794936 10.1105/tpc.114.134585 PMC4558658

[9] Zhang X , Feng C, Wang M et al. Plasma membrane-localized SlSWEET7a and SlSWEET14 regulate sugar transport and storage in tomato fruits. Hortic Res. 2021;8:186 34333539 10.1038/s41438-021-00624-w PMC8325691

[10] Klemens PAW , Patzke K, Deitmer J et al. Overexpression of the vacuolar sugar carrier *AtSWEET16* modifies germination, growth, and stress tolerance in *Arabidopsis*. Plant Physiol. 2013;163:1338–52 24028846 10.1104/pp.113.224972 PMC3813654

[11] Chardon F , Bedu M, Calenge F et al. Leaf fructose content is controlled by the vacuolar transporter SWEET17 in *Arabidopsis*. Curr Biol. 2013;23:697–702 23583552 10.1016/j.cub.2013.03.021

[12] Keller R , Ziegler C, Schneider D. When two turn into one: evolution of membrane transporters from half modules. Biol Chem. 2014;395:1379–88 25296672 10.1515/hsz-2014-0224

[13] Xu Y , Tao Y, Cheung LS et al. Structures of bacterial homologues of SWEET transporters in two distinct conformations. Nature. 2014;515:448–52 25186729 10.1038/nature13670 PMC4300204

[14] Lee Y , Nishizawa T, Yamashita K et al. Structural basis for the facilitative diffusion mechanism by SemiSWEET transporter. Nat Commun. 2015;6:6112 25598322 10.1038/ncomms7112 PMC4309421

[15] Xuan YH , Hu YB, Chen LQ et al. Functional role of oligomerization for bacterial and plant SWEET sugar transporter family. Proc Natl Acad Sci USA. 2013;110:E3685–94 24027245 10.1073/pnas.1311244110 PMC3785766

[16] Feng L , Frommer WB. Structure and function of SemiSWEET and SWEET sugar transporters. Trends Biochem Sci. 2015;40:480–6 26071195 10.1016/j.tibs.2015.05.005

[17] Guo WJ , Nagy R, Chen HY et al. SWEET17, a facilitative transporter, mediates fructose transport across the tonoplast of *Arabidopsis* roots and leaves. Plant Physiol. 2014;164:777–89 24381066 10.1104/pp.113.232751 PMC3912105

[18] Selvam B , Yu Y-C, Chen L-Q et al. Molecular basis of the glucose transport mechanism in plants. ACS Cent Sci. 2019;5:1085–96 31263768 10.1021/acscentsci.9b00252 PMC6598156

[19] Wang J , Yan C, Li Y et al. Crystal structure of a bacterial homologue of SWEET transporters. Cell Res. 2014;24:1486–9 25378180 10.1038/cr.2014.144 PMC4260346

[20] Lin IW , Sosso D, Chen LQ et al. Nectar secretion requires sucrose phosphate synthases and the sugar transporter SWEET9. Nature. 2014;508:546–9 24670640 10.1038/nature13082

[21] Wang J , Xue X, Zeng H et al. Sucrose rather than GA transported by AtSWEET13 and AtSWEET14 supports pollen fitness at late anther development stages. New Phytol. 2022;236:525–37 35811428 10.1111/nph.18368 PMC9795879

[22] Guan YF , Huang XY, Zhu J et al. RUPTURED POLLEN GRAIN1, a member of the MtN3/saliva gene family, is crucial for exine pattern formation and cell integrity of microspores in *Arabidopsis*. Plant Physiol. 2008;147:852–63 18434608 10.1104/pp.108.118026 PMC2409014

[23] Han L , Zhu Y, Liu M et al. Molecular mechanism of substrate recognition and transport by the AtSWEET13 sugar transporter. Proc Natl Acad Sci USA. 2017;114:10089–94 28878024 10.1073/pnas.1709241114 PMC5617298

[24] Sun MX , Huang XY, Yang J et al. *Arabidopsis* RPG1 is important for primexine deposition and functions redundantly with RPG2 for plant fertility at the late reproductive stage. Plant Reprod. 2013;26:83–91 23686221 10.1007/s00497-012-0208-1

[25] Gautam T , Dutta M, Jaiswal V. et al. Emerging roles of SWEET sugar transporters in plant development and abiotic stress responses. Cell. 2022;11:1303 10.3390/cells11081303 PMC9031177 35455982

[26] Braun DM . Phloem loading and unloading of sucrose: what a long, strange trip from source to sink. Annu Rev Plant Biol. 2022;73:553–84 35171647 10.1146/annurev-arplant-070721-083240

[27] Isoda R , Palmai Z, Yoshinari A et al. SWEET13 transport of sucrose, but not gibberellin, restores male fertility in *Arabidopsis* sweet13;14. Proc Natl Acad Sci USA. 2022;119:e2207558119 36215460 10.1073/pnas.2207558119 PMC9586311

[28] Fakher B , Jakada BH, Greaves JG. et al. Identification and expression analysis of pineapple sugar transporters reveal their role in the development and environmental response. Front Plant Sci. 2022;13:964897 10.3389/fpls.2022.964897 PMC9638087 36352877

[29] Wu LB , Eom JS, Isoda R et al. OsSWEET11b, a potential sixth leaf blight susceptibility gene involved in sugar transport-dependent male fertility. New Phytol. 2022;234:975–89 35211968 10.1111/nph.18054

[30] Breia R , Conde A, Badim H et al. Plant SWEETs: from sugar transport to plant-pathogen interaction and more unexpected physiological roles. Plant Physiol. 2021;186:836–52 33724398 10.1093/plphys/kiab127 PMC8195505

[31] Eom JS , Chen LQ, Sosso D et al. SWEETs, transporters for intracellular and intercellular sugar translocation. Curr Opin Plant Biol. 2015;25:53–62 25988582 10.1016/j.pbi.2015.04.005

[32] Ji J , Yang L, Fang Z. et al. Plant SWEET family of sugar transporters: structure, evolution and biological functions. Biomol Ther. 2022;12:205 10.3390/biom12020205 PMC8961523 35204707

[33] Chen LQ , Cheung LS, Feng L et al. Transport of sugars. Annu Rev Biochem. 2015;84:865–94 25747398 10.1146/annurev-biochem-060614-033904

[34] Tao Y , Cheung LS, Li S et al. Structure of a eukaryotic SWEET transporter in a homotrimeric complex. Nature. 2015;527:259–63 26479032 10.1038/nature15391 PMC4734654

[35] Yamada K , Osakabe Y, Yamaguchi-Shinozaki K. A C-terminal motif contributes to the plasma membrane localization of *Arabidopsis* STP transporters. PLoS One. 2017;12:e0186326 29028820 10.1371/journal.pone.0186326 PMC5640241

[36] Nobukuni M , Mochizuki H, Okada S et al. The C-terminal region of serotonin transporter is important for its trafficking and glycosylation. J Pharmacol Sci. 2009;111:392–404 19946177 10.1254/jphs.09195fp

[37] Fujita S , Sato D, Kasai H et al. The C-terminal region of the yeast monocarboxylate transporter Jen1 acts as a glucose signal-responding degron recognized by the α-arrestin Rod1. J Biol Chem. 2018;293:10926–36 29789424 10.1074/jbc.RA117.001062 PMC6052204

[38] Tamura K , Stecher G, Peterson D et al. MEGA6: Molecular Evolutionary Genetics Analysis version 6.0. Mol Biol Evol. 2013;30:2725–9 24132122 10.1093/molbev/mst197 PMC3840312

[39] Kumar S , Stecher G, Tamura K. MEGA7: Molecular Evolutionary Genetics Analysis version 7.0 for bigger datasets. Mol Biol Evol. 2016;33:1870–4 27004904 10.1093/molbev/msw054 PMC8210823

[40] Omasits U , Ahrens CH, Müller S et al. Protter: interactive protein feature visualization and integration with experimental proteomic data. Bioinformatics. 2014;30:884–6 24162465 10.1093/bioinformatics/btt607

[41] Jumper J , Evans R, Pritzel A et al. Highly accurate protein structure prediction with AlphaFold. Nature. 2021;596:583–9 34265844 10.1038/s41586-021-03819-2 PMC8371605

[42] Clough SJ , Bent AF. Floral dip: a simplified method for *Agrobacterium*-mediated transformation of *Arabidopsis thaliana*. Plant J. 1998;16:735–43 10069079 10.1046/j.1365-313x.1998.00343.x

[43] Aslam M , Fakher B, Jakada BH. et al. Genome-wide identification and expression profiling of CBL-CIPK gene family in pineapple (*Ananas comosus*) and the role of AcCBL1 in abiotic and biotic stress response. Biomol Ther. 2019;9:293 10.3390/biom9070293 PMC6681290 31330847

[44] Wang L , Li Y, Jin X et al. Floral transcriptomes reveal gene networks in pineapple floral growth and fruit development. Commun Biol. 2020;3:500 32913289 10.1038/s42003-020-01235-2 PMC7483743

[45] Livak KJ , Schmittgen TD. Analysis of relative gene expression data using real-time quantitative PCR and the 2(-Delta Delta C(T)) method. Methods. 2001;25:402–8 11846609 10.1006/meth.2001.1262

[46] Bashandy H , Jalkanen S, Teeri TH. Within leaf variation is the largest source of variation in agroinfiltration of *Nicotiana benthamiana*. Plant Methods. 2015;11:47 26472987 10.1186/s13007-015-0091-5 PMC4607171

[47] Peterson. R , Slovin J, Chen C. A simplified method for differential staining of aborted and non-aborted pollen grains. Int J Plant Biol. 2010;1:e13

[48] Karimi M , Inzé D, Depicker A. GATEWAY™ vectors for *Agrobacterium*-mediated plant transformation. Trends Plant Sci. 2002;7:193–5 11992820 10.1016/s1360-1385(02)02251-3

[49] Wieczorke R , Krampe S, Weierstall T et al. Concurrent knock-out of at least 20 transporter genes is required to block uptake of hexoses in *Saccharomyces cerevisiae*. FEBS Lett. 1999;464:123–8 10618490 10.1016/s0014-5793(99)01698-1

[50] Guo C , Li H, Xia X et al. Functional and evolution characterization of SWEET sugar transporters in Ananas comosus. Biochem Biophys Res Commun. 2018;496:407–14 29307830 10.1016/j.bbrc.2018.01.024

