# Supplementary material for: Pineapple SWEET10 is a glucose transporter
Source: Hortic Res. 2023 Apr 12;10(10):uhad175. doi: 10.1093/hr/uhad175 (PMC10660354; doi:10.1093/hr/uhad175)
Supplement: Web_Material_uhad175 [file web_material_uhad175.zip › Figure S1.pdf]

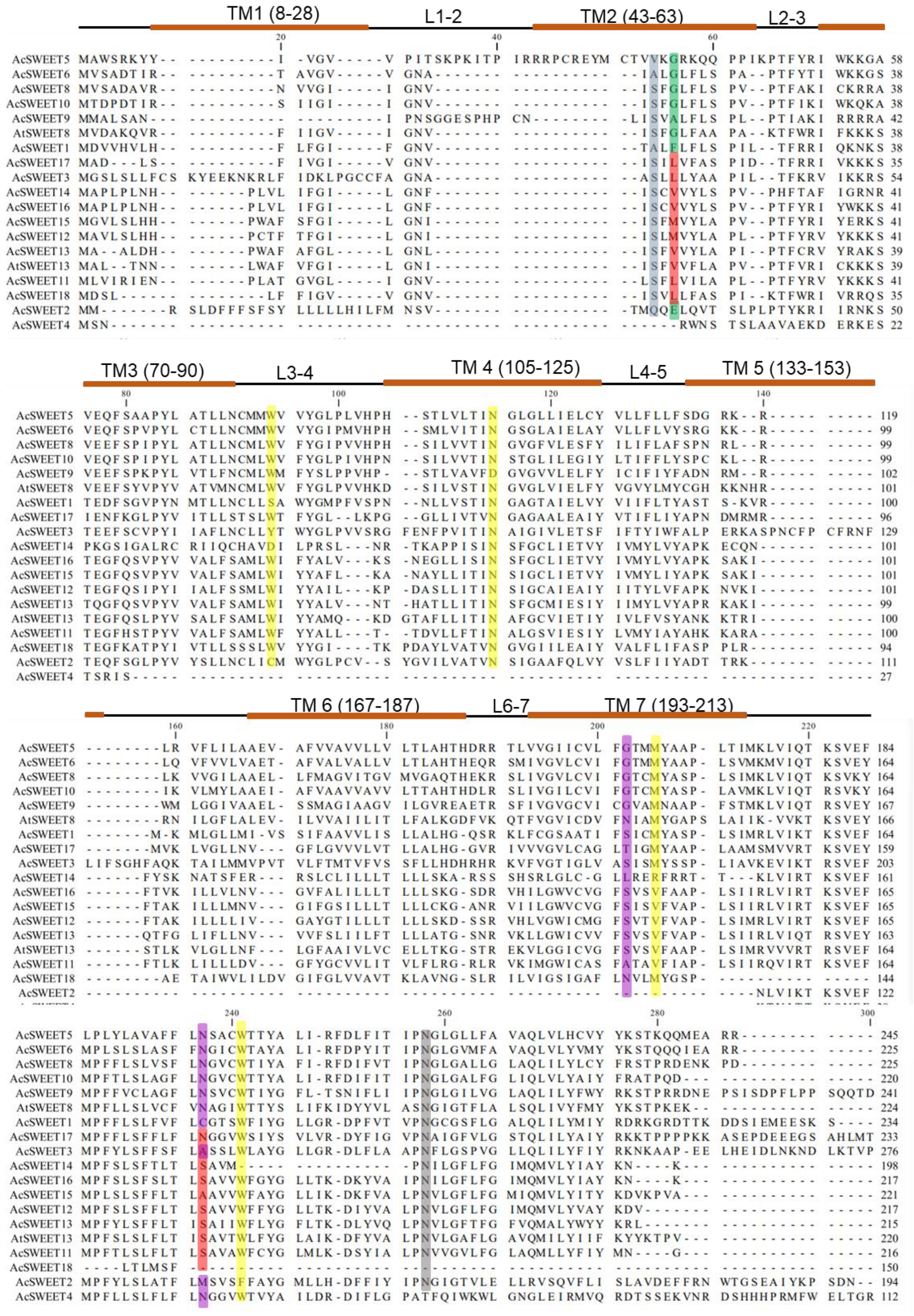

Figure S1: Multiple sequence alignments of SWEET proteins of pineapple. AtSWEET8 and AtSWEET13 serve as the reference. The numbers on top of the table represent AtSWEET13.
