# Supplementary material for: Pineapple SWEET10 is a glucose transporter
Source: Hortic Res. 2023 Apr 12;10(10):uhad175. doi: 10.1093/hr/uhad175 (PMC10660354; doi:10.1093/hr/uhad175)
Supplement: Web_Material_uhad175 [file web_material_uhad175.zip › Figure S2.pdf]

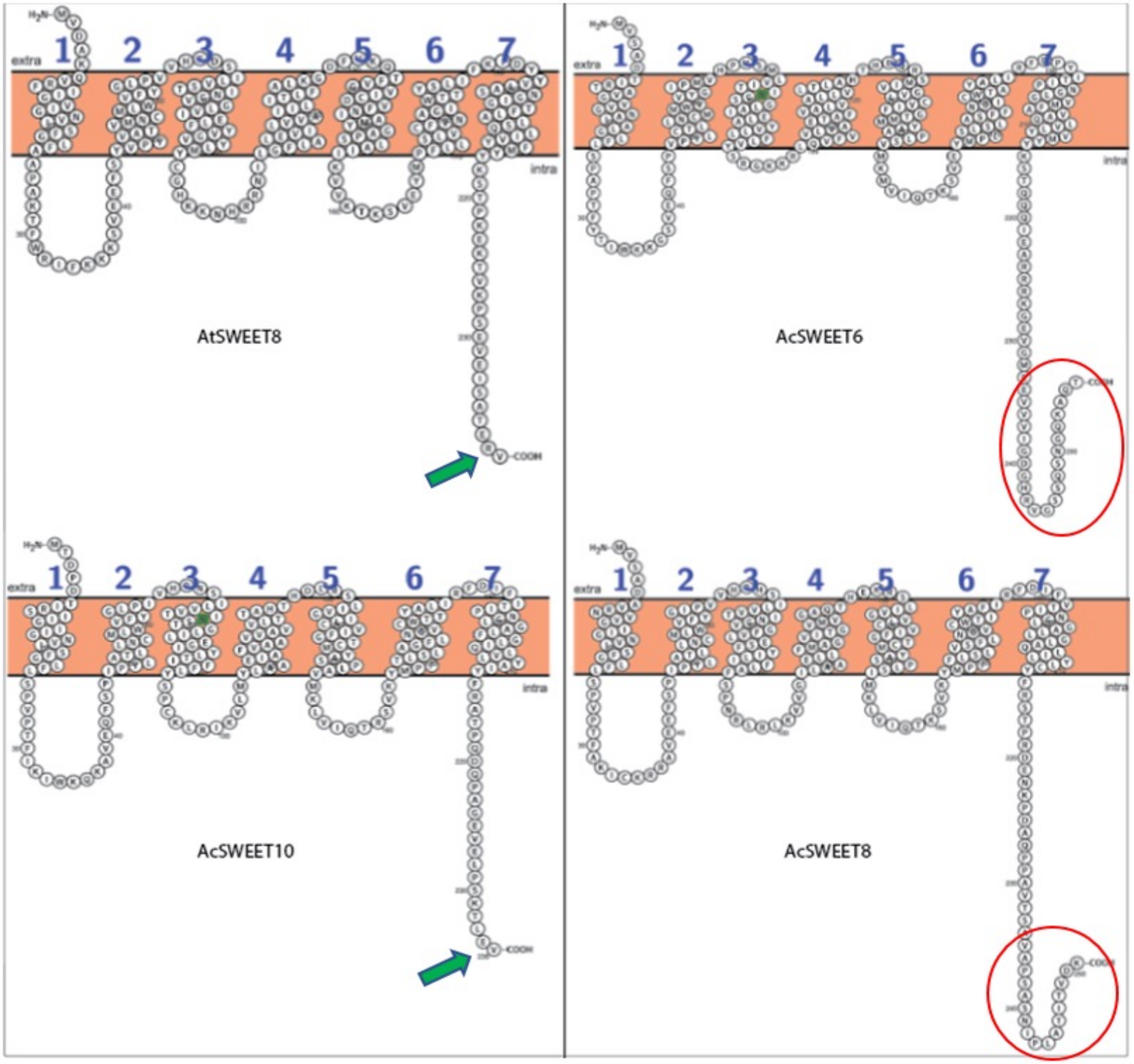

Figure S2: Predicted transmembrane topology of AcSWEET6, AcSWEET8, AcSWEET10 and AtSWEET8 by the Protter protein-visualization tool. Protein sequences shows seven transmembrane helices. Arrows (green) and circles (red) represent similarity and dissimilarity in C-terminal ends, respectively.
