# Supplementary material for: Pineapple SWEET10 is a glucose transporter
Source: Hortic Res. 2023 Apr 12;10(10):uhad175. doi: 10.1093/hr/uhad175 (PMC10660354; doi:10.1093/hr/uhad175)
Supplement: Web_Material_uhad175 [file web_material_uhad175.zip › Figure S3&S4.pdf]

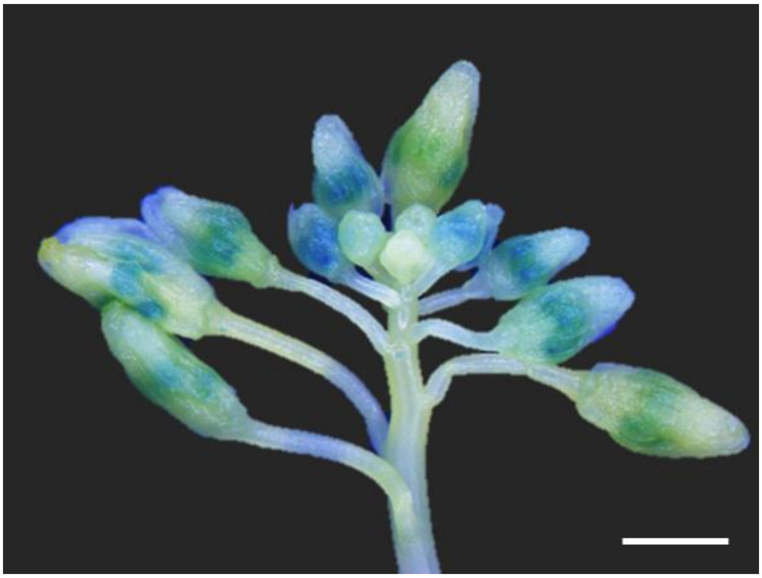

Figure S3: Histochemical GUS localization of AtSWEET8 in which GUS expression was driven with the SWEET8 promoter with the *SWEET8* gene. Three independent lines were analyzed with similar results. Scale bar: 1 mm

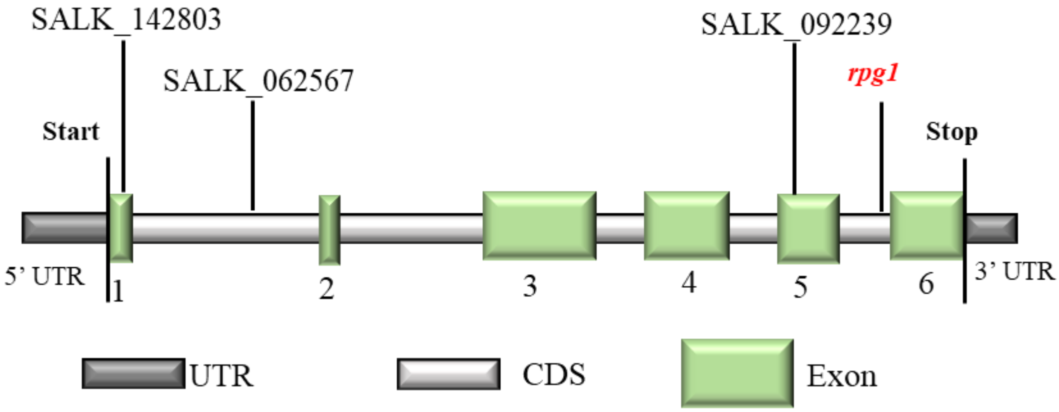

Figure S4: Schematic representation of T-DNA insertion sites in the *SWEET8* gene of *Arabidopsis*.
