# Supplementary material for: Pineapple SWEET10 is a glucose transporter
Source: Hortic Res. 2023 Apr 12;10(10):uhad175. doi: 10.1093/hr/uhad175 (PMC10660354; doi:10.1093/hr/uhad175)
Supplement: Web_Material_uhad175 [file web_material_uhad175.zip › Supplementary Table S2.pdf]

Table S2: Physicochemical properties of pineapple SWEETs and AtSWEET8.

Gene name	Gene ID	Length (aa)	MW(Da)	pI	I.I.	A.I.	GRAVY	Sub-loc.	TMH
AcSWEET1	Aco011302	258	28.902	9.21	34.93	107.64	0.519	PM	7
AcSWEET2	Aco016508	194	22.292	7.67	32.41	114.48	0.664	PM	3
AcSWEET3	Aco010708	288	32.685	9.34	46.28	106.98	0.45	PM	7
AcSWEET4	Aco006346	175	20.285	8.47	42.09	75.77	-0.393	Chlo	1
AcSWEET5	Aco005793	288	32.614	9.75	44.63	113.68	0.445	PM	6
AcSWEET6	Aco004463	256	28.143	9.16	39.03	113.75	0.654	PM	6
AcSWEET7	Aco006158	885	98.972	9.47	45.09	96.84	0.006	PM	8
AcSWEET8	Aco006156	251	27.396	9.2	42.77	124.98	0.885	PM	7
AcSWEET9	Aco006155	423	45.779	5.29	71.79	95.04	0.192	PM	7
AcSWEET10	Aco016418	235	25.985	8.58	41.55	132.3	0.927	PM	7
AcSWEET11	Aco001900	268	29.775	9.01	37.7	121.12	0.703	PM	7
AcSWEET12	Aco019048	281	31.573	8.93	40.49	121	0.577	PM	7
AcSWEET13	Aco004628	274	30.92	7.66	43.75	124.45	0.777	PM	7
AcSWEET14	Aco016039	283	31.731	9.19	56.81	94.38	-0.068	V	3
AcSWEET15	Aco003627	265	29.65	9.71	27.51	127.25	0.773	PM	7
AcSWEET16	Aco017831	302	33.36	6.53	40.34	118.34	0.549	PM	7
AcSWEET17	Aco002476	293	32.113	9.5	31.97	116.08	0.499	V	7
AcSWEET18	Aco006347	150	16.29	9.4	32.62	143.53	1.155	V	4
AtSWEET8	At5G40260	240	26.8	9.6	41	121.38	0.764	PM	7

PM- plasma membrane; Chlo- chloroplasts; V-Vacuole

*Legends: aa -amino acid; MW- molecular weight in daltons (Da); pI- isoelectric point; I.I. -instability index; GRAVY- grand average of hydropathy; Loc- subcellular localization; TMH- transmembrane helices.
