# Supplementary material for: Pineapple SWEET10 is a glucose transporter
Source: Hortic Res. 2023 Apr 12;10(10):uhad175. doi: 10.1093/hr/uhad175 (PMC10660354; doi:10.1093/hr/uhad175)
Supplement: Web_Material_uhad175 [file web_material_uhad175.zip › Supplementary Table S3.pdf]

Table S3: List of the primers used in the study.

Name	Primer Sequence (5'---> 3')	
	Forward	Reverse
**A. RT-qPCR**		
AtSWEET8	GTGGTGGCTATCATTCTTAT	TCCAGCATTAACGAAACAGA
AcSWEET5	CCTATGCTCTCATCCGCTTC	CCTCTGCCAGACTCAACTCC
AcSWEET6	CACCAGCGCCAACTTTTTAT	ACGAGGAAGAGGAGCACGTA
AcSWEET7	AGCAGCAGCAGCAGTTACAA	CATGCTTCAATTGCTCCTCA
AcSWEET8	GGTGATTACTGGGGTGATGG	CGGATGAAGGCATAGATCGT
AcSWEET9	CCTACCTCGTGACGCTCTTC	ACGACAACTCCGCAGCTACT
AcSWEET10	CTGGACCACCTATGCCCTAA	CAAGAGTCTTCGAGGGCAAC
AcEF1a	TCTTCTCAGGGAAGGTCTCTAC	CTCTGCACACTCTTCACATACA
**B. Cloning**		
AtSWEET8	CACCATGGTTGATGCAAAACAAGT	AACCCTCTCCGTAGCAGAAAT
AcSWEET6	CACCATGGTTTCTGCTGATACCATC	GGTTTGAGCTTTTTGGCC
AcSWEET8	CACCATGGTCTCCGCCGACGCAGTT	TTTATCTACTGTGATAGTGGC
AcSWEET10	CACCATGACTGATCCTGATACTATCC	GACTTCAAGAGTCTTCGAGG
**C. Yeast complementation assay**		
AtSWEET8	ATGGTTGATGCAAAACAAGTTCGT	AACCCTCTCCGTAGCAGAAAT
AcSWEET6	ATGGTTTCTGCTGATACCATC	GGTTTGAGCTTTTTGGCC
AcSWEET8	ATGGTCTCCGCCGACGCAGTT	TTTATCTACTGTGATAGTGGC
AcSWEET10	ATGACTGATCCTGATACTATCC	GACTTCAAGAGTCTTCGAGG
