# Supplementary material for: Pineapple SWEET10 is a glucose transporter
Source: Hortic Res. 2023 Apr 12;10(10):uhad175. doi: 10.1093/hr/uhad175 (PMC10660354; doi:10.1093/hr/uhad175)
Supplement: Web_Material_uhad175 [file web_material_uhad175.zip › SupplementaryTable S1.pdf]

Table S1: Pairwise comparison of the amino acid identity of Pineapple SWEET (18), AtSWEET13, AtSWEET8 and OsSWEET2b.

	AcSWEET4	AtSWEET8	AcSWEET9	AcSWEET7	AcSWEET5	AcSWEET6	AcSWEET8	AcSWEET10	AcSWEET11	AtSWEET13	AcSWEET13	AcSWEET14	AcSWEET12	AcSWEET15	AcSWEET16	AcSWEET3	AcSWEET17	AcSWEET18	AcSWEET2	AcSWEET1	AcSWEET2b
1: AcSWEET4		27.59	23.42	26.09	27.10	28.28	32.63	31.82	23.14	18.11	23.01	27.18	25.49	23.64	25.41	25.74	25.19	10.34	32.35	26.61	26.14
2: AtSWEET8			33.48	38.46	39.57	44.78	44.35	43.67	34.84	33.04	33.48	20.69	32.58	32.00	33.48	34.06	39.64	35.33	30.99	39.37	30.63
3: AcSWEET9				32.95	34.32	37.25	54.80	45.30	26.64	25.70	27.27	19.12	26.67	26.09	26.90	28.80	29.62	28.77	31.98	32.53	34.55
4: AcSWEET7					40.16	45.81	67.12	57.77	29.54	25.31	25.33	18.14	27.95	25.44	25.00	25.55	30.65	36.81	24.84	33.04	27.27
5: AcSWEET5						60.16	47.81	55.32	29.60	26.40	27.16	19.74	29.79	27.89	28.74	29.96	32.39	32.43	31.11	35.08	32.89
6: AcSWEET6							54.98	60.00	32.47	31.65	32.30	24.40	36.04	31.90	35.96	30.33	38.03	36.11	33.14	40.95	37.44
7: AcSWEET8								68.09	34.36	34.33	32.44	24.76	34.23	32.75	35.11	32.08	41.48	37.50	31.95	43.17	36.53
8: AcSWEET10									36.74	34.39	33.02	27.04	34.88	34.25	37.67	31.58	43.78	38.19	34.52	43.78	37.61
9: AcSWEET11										47.33	51.38	29.17	52.89	50.00	48.65	31.84	36.78	36.91	31.21	33.20	31.25
10: AtSWEET13											52.59	30.30	51.89	54.05	51.06	30.92	35.94	36.00	30.46	36.14	34.22
11: AcSWEET13												34.65	56.86	58.75	58.39	33.19	32.20	33.56	29.76	31.67	31.05
12: AcSWEET14													43.68	43.57	65.25	23.42	24.90	14.48	23.87	21.49	20.30
13: AcSWEET12														72.06	63.70	36.21	38.22	38.26	32.35	37.13	31.67
14: AcSWEET15															71.65	34.55	37.40	35.57	32.76	36.18	30.67
15: AcSWEET16																30.99	35.36	32.89	34.12	36.03	34.39
16: AcSWEET3																	34.43	32.67	38.71	39.75	37.99
17: AcSWEET17																		52.67	34.71	43.03	36.49
18: AcSWEET18																			31.25	39.19	33.11
19: AcSWEET2																				44.25	50.00
20: AcSWEET1																					45.18
21: OsSWEET2b																					
