# Supplementary figures and images for: Pineapple SWEET10 is a glucose transporter
Source: Hortic Res. 2023 Apr 12;10(10):uhad175. doi: 10.1093/hr/uhad175 (PMC10660354; doi:10.1093/hr/uhad175)

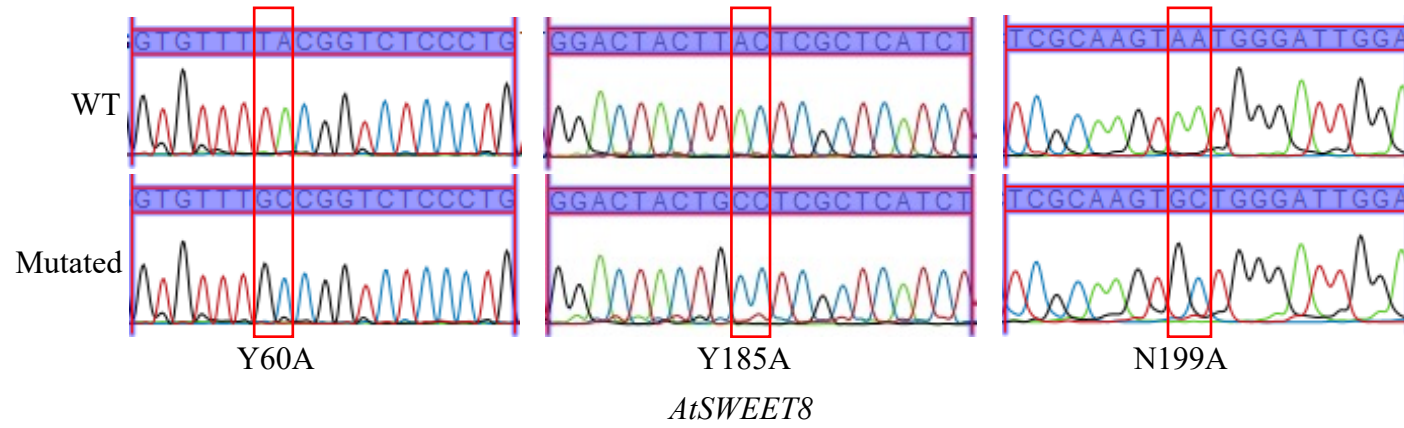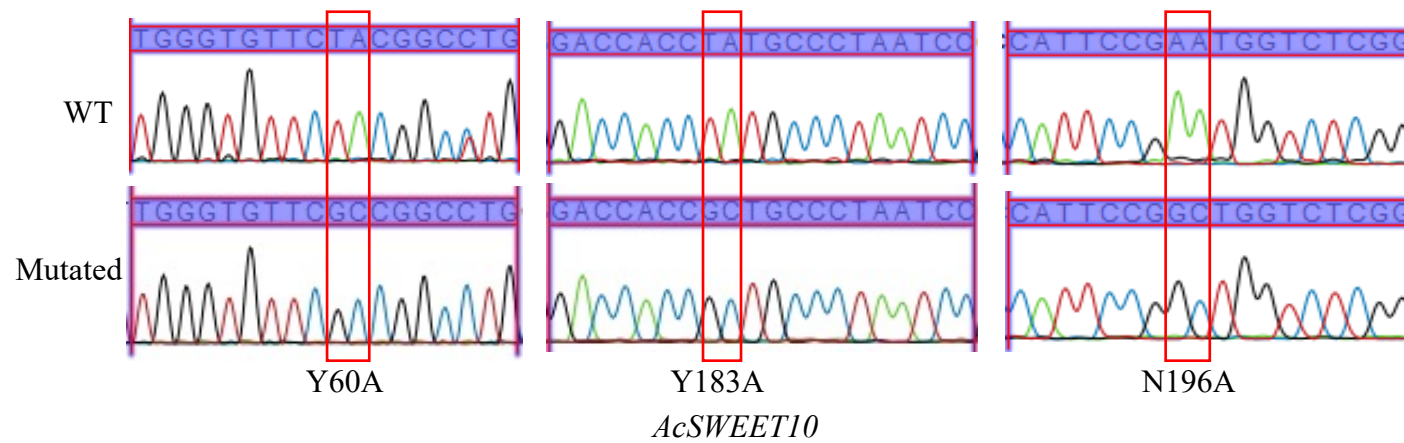

Supplement: Web_Material_uhad175 [file web_material_uhad175.zip › Figure S5.pdf]

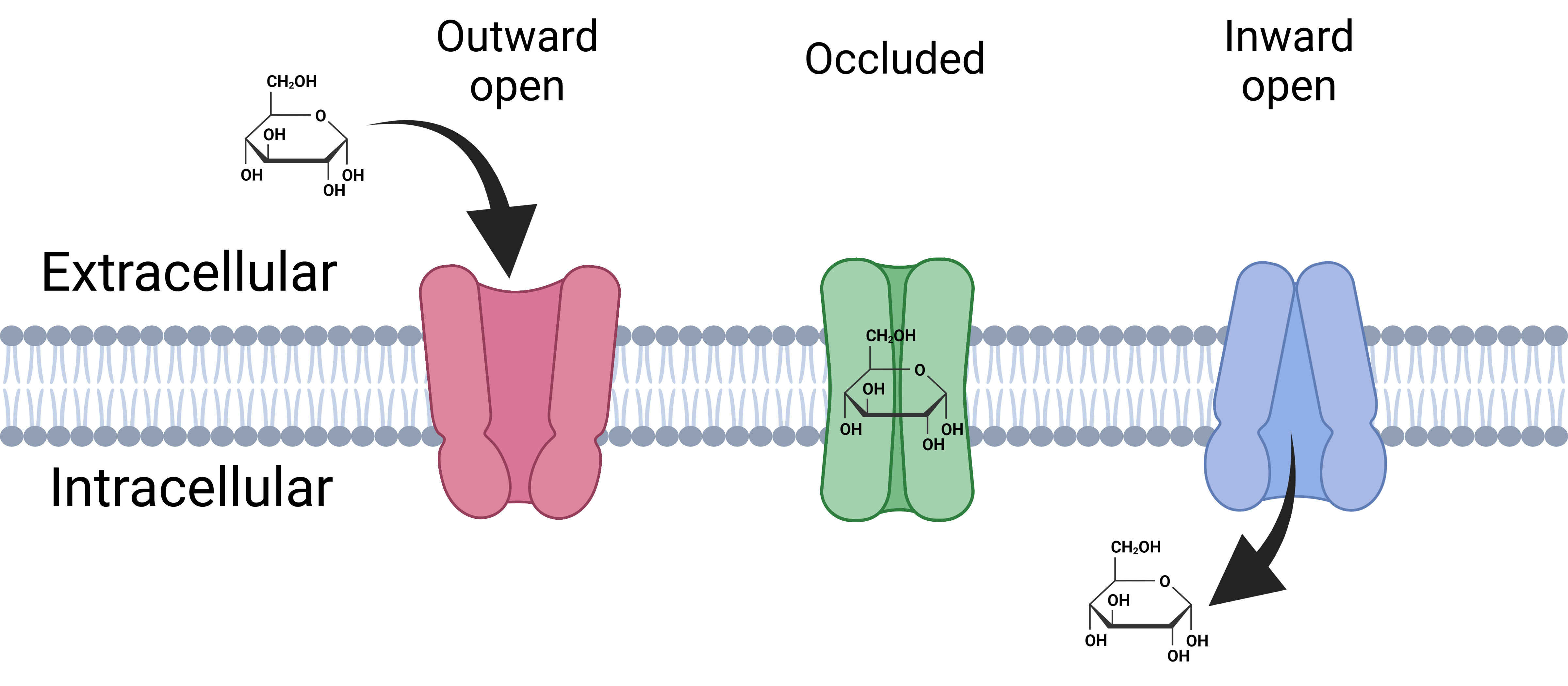

Supplement: Web_Material_uhad175 [file web_material_uhad175.zip › Figure S6.jpg]
